# Universal infant immunisation against respiratory syncytial virus and European inequalities: the pandemics lesson has not been learnt

**DOI:** 10.1016/j.lanepe.2023.100753

**Published:** 2023-10-11

**Authors:** Daniele De Luca, Manuel Sanchez-Luna, Karl Schettler, Louis Bont, Eugenio Baraldi

**Affiliations:** a Division of Paediatrics and Neonatal Critical Care, “Antoine Béclère” Medical Centre, Paris Saclay University Hospitals, APHP, Paris, France; b Physiopathology and Therapeutic Innovation Unit-INSERM U999, Paris Saclay University, Paris, France; c Division of Neonatology, University Hospital Gregorio Marañon Madrid, Complutense University, Spain; d Division of Paediatric and Neonatal Intensive Care, Children's Hospital St. Marien GmbH, Landshut, Germany; e Department of Paediatrics, University Medical Centre Utrecht, the Netherlands; f Respiratory Syncytial Virus Network (RESVINET) Foundation, Zeist, the Netherlands; g Neonatal Intensive Care Unit, Department of Woman's and Child's Health, University Hospital of Padova, Padua, Italy

Respiratory syncytial virus (RSV) is the commonest cause of respiratory infection in the early infancy^1^ and, in severest cases, it may trigger acute respiratory distress syndrome.^2^^,^^3^ It often requires oxygen supplementation and respiratory support: this translates in several days of hospital stay with relevant costs. RSV is highly contagious and, besides the burden on the family (e.g.: parental anxiety and lost workdays), during the winter season, the patients’ need can overwhelm the hospital resources. In fact, the admissions to European paediatric (PICU) and neonatal intensive care unit (NICU) recently increased4^4^ and PICU/NICU beds have not been sufficient in some areas with infants being transferred hundreds of kilometres away from home. This received wide media coverage and reached the titles in major scientific journals (“*RSV hammers the hospitals*”).^5^

This makes RSV infection a public health problem reproducing what we have recently witnessed during pandemics, on a larger scale, for adult critical care. However, RSV seasonality makes the problem serial as the virus easily infects children who have not encountered it in the previous season. This year major game changers, such as maternal vaccination and nirsevimab, were approved by regulatory agencies, including the European Medicine Agency (EMA).^6^ Nirsevimab is a recombinant monoclonal antibody binding the fusion protein of RSV,^7^ and one injection was shown to provide long-lasting protection with decreased hospitalization of term and preterm infants.^8^ This paved the way to an universal immunisation, that was previously unfeasible with palivizumab. This was a different monoclonal antibody needing monthly injections and was reserved only to high-risk patients (e.g.: former preterm infants and those affected by relevant comorbidities).

From the public health perspective, an important benefit of the universal immunisation is the preservation of PICU/NICU beds. This is critical because, while RSV infections are seasonal, other common causes of PICU/NICU admission are not and represent an unpredictable demand. Therefore, the burden induced by RSV season may have a negative impact on the quality of care, leaving no beds for children requiring critical care to survive. The “protection” of PICU/NICU beds is an important concept that was highlighted even during SARS-CoV-2 pandemics with the same aim, that is, guaranteeing the availability and quality of PICU/NICU care.^9^

Nonetheless, we are facing a panoply of different positions across EU countries: despite the EMA approval, the immunisation is progressing at different paces and only few countries have started the campaign (see Fig. 1). In most countries, the immunisation is unlikely to start before the incoming winter. This is creating worrisome disparities and may jeopardize the achievement of a population immunity and the preservation of PICU/NICU beds for the most critically ill patients. This is particularly important given the limited resources for public healthcare, which represent a problem shared by several countries. On the other hand, an even worse delay is observed in low- and middle-income countries where the mortality is unacceptably high: thus, for the immunisation to exploit its full potential, all barriers to a capillary and uniform campaign shall be removed.^10^

**Fig. 1:**
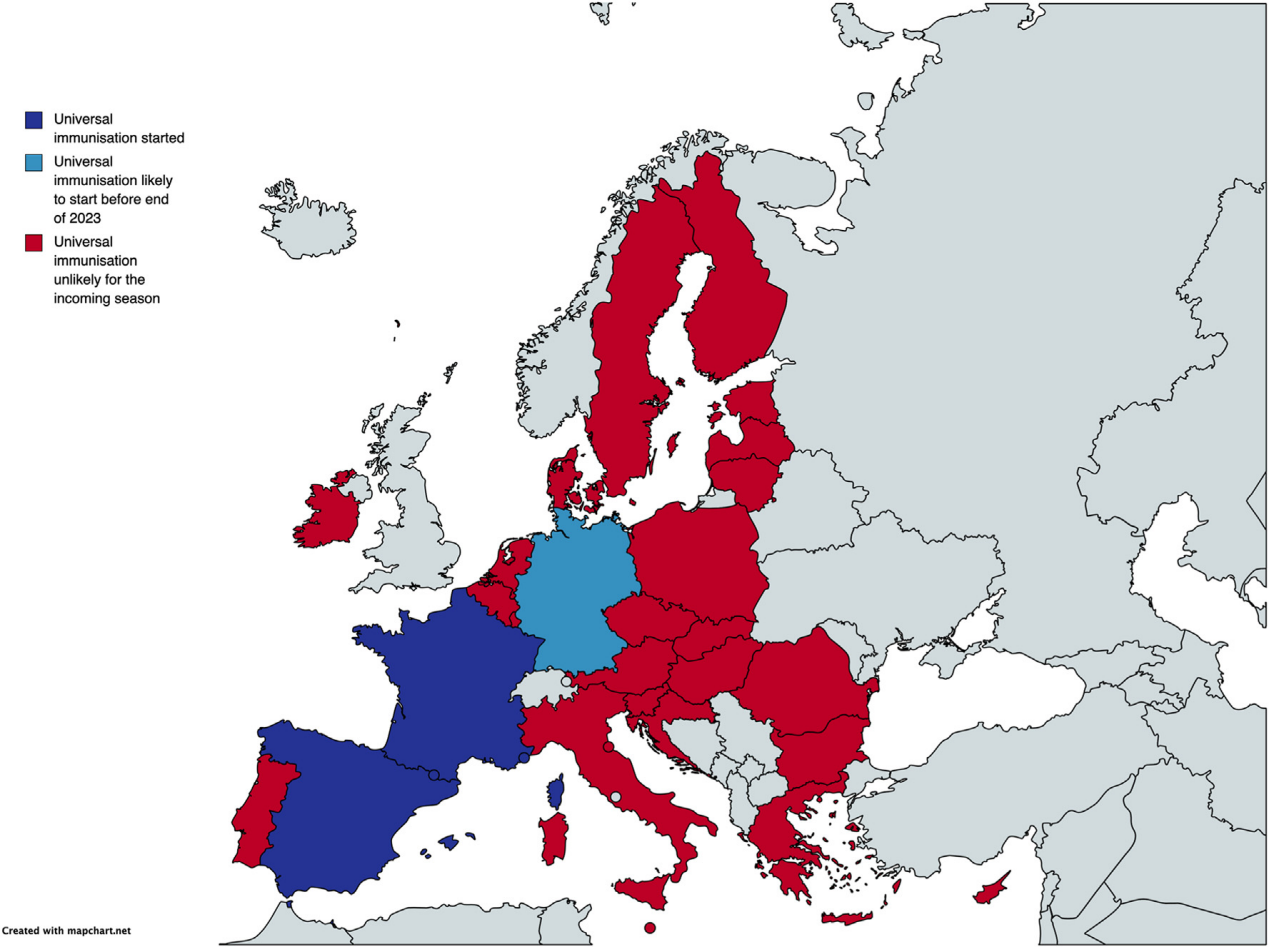
**Diffusion of universal RSV immunisation in European Union countries**. Data obtained by contacting directly local clinical key opinion leaders or authorities (personal communications updated at October 3, 2023).

The reasons for such delay are various. Some countries have an excessively regionalised healthcare system which responds inefficiently to generalised emergencies such as outbreaks. These systems are less prepared for the implementation of an universal, seasonal, large-scale immunization: therefore, relevant decisions in this field should be completely taken on a nation- or, even better, continent-wide level, to be more quickly and efficaciously implemented. Some European countries have additional local procedures that may delay the immunisation campaign. This is happening even in EU countries where the EMA approval already stands, and local regulatory steps may represent complex bureaucratic complications. Some countries may want to wait a cost-effectiveness analysis which is indeed difficult to conduct in this domain, as some costs (e.g.: PICU/NICU lost beds, parental anxiety and lost workdays) are difficult to estimate. Finally, as RSV outbreak is around the corner, the implementation of immunisation should have followed a dedicated urgent pathway rather than that of any other drug. However, local authorities have not given nirsevimab the same urgency attributed to SARS-CoV-2 vaccines. In fact, delaying such an urgent intervention is more critical than postponing the introduction of a drug unrelated to a seasonal problem. No doubts, these issues are also partially related to a relatively low attention by our paediatric community: nonetheless, the problem shall not be underestimated for its public health consequences.

We need to remove the aforementioned barriers: this would be a significant step forward for European infants and a good example to diffuse immunisation also in low- and middle-income countries. Meanwhile, we must acknowledge that the lesson taught by pandemics has not been learnt and this now endangers infants in Europe.

## Contributors

DDL conceived the manuscript, wrote the first draft and collected the information on the immunisation campaign. MSL, KS and LB contributed to literature analysis, collection of information and critically contributed to the manuscript for important intellectual contents. EB collected information on the immunisation campaign, performed the literature search and review and critically contributed to the manuscript for important intellectual contents. All authors approved the final manuscript version.

## Editor note

The Lancet Group takes a neutral position with respect to territorial claims in published maps and institutional affiliations.

## Declaration of interests

DDL received consultancy and lecture fees from Chiesi Farmaceutici, Getinge, Vyaire, Radiometer, Medtronic, Astra Zeneca, Boehringer Ingelheim, Airway Therapeutics, Natus, Masimo; he also has equity options from Ophirex ltd. All these were unrelated to the present work and to nirsevimab and the universal RSV immunisation. MSL received consultancy and lecture fees from Medtronic, Astra Zeneca, and Sanofi, all outside of the present work and unrelated to nirsevimab and the universal RSV immunisation. EB received consultancy and lecture fees and participated to advisory boards for Astra-Zeneca and Sanofi, all outside of the present work. LB is the founding chairman of the ReSViNET Foundation. He has not received any personal benefit. His institution has conversely received research grants from AbbVie, MedImmune, AstraZeneca, Sanofi, Janssen, Pfizer, MSD and MeMed Diagnostics, GSK and Novavax, Julius Clinical. His institution also received consultancy and invited lecture fees by AbbVie, MedImmune, Ablynx, Bavaria Nordic, MabXience, GSK, Novavax, Pfizer, Moderna, Astrazeneca, MSD, Sanofi and Janssen. All these were unrelated to the present work. KS has no conflict of interest to declare.

This work did not receive any funding.
